# Differences in event-related potentials between unipolar depression and bipolar II disorder during depressive episodes: a retrospective case-control study

**DOI:** 10.1186/s12888-025-07433-8

**Published:** 2025-10-22

**Authors:** Xiaobo Zhou, Jingwen Liu, Zhonghua Lin, Minjing Xiang, Xia Deng, Zhili Zou

**Affiliations:** 1 Provincial Center for Mental Health, Sichuan Provincial People’s Hospital, University of Electronic Science and Technology of China, 32# W. Sec 2, 1St Ring Rd. Qingyang District, Chengdu, 610000 China; 2 Department of Psychosomatic Medicine, Sichuan Provincial People’s Hospital, University of Electronic Science and Technology of China, Chengdu, 610000 China

**Keywords:** Bipolar II disorder, Unipolar depression, Event-related potential, Cognition, P300

## Abstract

**Background:**

Bipolar II disorder (BD II) is a chronic and severe mental illness frequently misdiagnosed as major depressive disorder (MDD) due to symptom overlap and the absence of objective diagnostic tools. Consequently, establishing pathophysiological markers to differentiate BD II from MDD is critical.

**Method:**

A total of 180 patients were enrolled in the study and allocated to three groups: patients with unipolar depression (UD group; MDD currently experiencing a major depressive episode, *n* = 60), patients with bipolar II disorder during depressive episodes (BD II group; *n* = 60), and age- and sex- matched healthy controls (HC; *n* = 60). Sociodemographic data were collected, and all participants underwent psychological assessments using the 7-item Generalized Anxiety Disorder (GAD-7), Patient Health Questionnaire-9 (PHQ-9), and 32-item Hypomania Checklist (HCL-32). Additionally, all participants passed auditory brain stem response (ABR) test and subsequently underwent event-related potential (ERP) examinations.

**Results:**

No significant differences were observed in demographic characteristics between the three groups, including age, sex, educational level, marital status, and socioeconomic status (all *P* > 0.05). Compared with HC, patients in both the UD and BD II groups showed significantly longer reaction time (HC: 254.4 ± 43.8 ms; UD: 297.7 ± 72.2 ms; BD II: 300.3 ± 70.0 ms; *P* = 0.028) and larger amplitude of P2-N2 complex (HC: 5.7 ± 4.4 μV; UD: 8.1 ± 4.8 μV; BD II: 8.6 ± 5.6 μV; *P* = 0.001) in P300 paradigm. The BD II group exhibited longer S2-P50 latency than the UD group (UD: 50.4 ± 11.1 ms vs. BD II: 63.2 ± 11.5 ms; *P* = 0.025). Additionally, the BD II group had prolonged N2 latency compared to HC (BD II: 216.2 ± 22.1 ms vs. HC: 205.2 ± 16.5 ms; *P* = 0.044).

**Conclusions:**

This study may identify neurophysiological distinctions between BD II and UD depression, notably a prolonged S2-P50 latency in BD II.

## Introduction

Bipolar II disorder (BD II) is a chronic and severe psychiatric condition characterized by recurrent major depressive episodes alternating with hypomanic episodes. A nationwide epidemiological study [1] reported a lifetime prevalence of 0.09% for bipolar I disorder and 0.04% for bipolar II disorder in China. Compared with bipolar I disorder, BD II is more likely to be misdiagnosed as unipolar depression in clinical settings [2]. Individuals with bipolar II disorder have a significantly higher lifetime suicide risk than the general population [3, 4]. Additionally, a number of studies have reported relatively higher rates of completed suicides in patients with BD II compared to BD I [5].

BD II is inherently diagnostically challenging, with the majority of patients initially presenting with depressive symptomatology. This diagnostic complexity contributes to substantial under-detection [6], with studies indicating that nearly 40% of BD-II cases are misclassified as major depressive disorder (MDD; also known as unipolar depression [UD]) during initial evaluations [7]. The resultant diagnostic uncertainty creates a perilous clinical gap – the median interval from first psychiatric consultation to correct BD II diagnosis exceeds 10 years [8]. The consequences of misdiagnosis of BD II include inappropriate treatment choices, such as under-prescription of mood-stabilizing medications, rapid cycling, relapse and hospitalization, increased risk of suicidal thoughts and behaviors, cognitive and functional impairments, and increased care expenses [8].

However, mental disorders pose complex diagnostic challenges, as current classifications, whether Diagnostic and Statistical Manual of Mental Disorders (DSM-V) or International Classification of Diseases (ICD-11), rely predominantly on symptomatology due to the lack of validated objective biomarkers. Compared to UD, earlier onset of depressive episodes and emotional instability may represent distinguishing clinical features of BD II. However, these features pose challenges for objectively quantifying distinctions between UD and BD II [9, 10]. Identifying robust neurophysiological or biochemical biomarkers to distinguish BD II from UD has proven highly challenging Despite promising accuracy of fMRI [Area under the curve (AUC): 0.84–0.94] [11] and gut microbiome genomic analysis (AUC: 0.72–0.88) [12] in differentiating BD II and UD, their clinical translation is constrained by practical high expense. Meanwhile, serum cytokine profiling exhibits only modest discriminatory capability (AUC: 0.69–0.74) [13], underscoring the need for cost-effective and reliable biomarkers like electrophysiological indices.

Event-related potentials (ERP) offer an objective, non-invasive, and economical method to detect neural activity associated with cognitive and sensory processes [14]. ERP comprise key components such as mismatch negativity (MMN), P300, contingent negative variation (CNV), and P50 suppression. Characteristics of these components, including presence, amplitude, topography, and latency, could serve as indicators of cognitive performance and provide objective measures for detecting changes in neurocognitive functioning associated with specific disorders [15, 16]. Many previous studies have shown impairments in psychosocial functioning in individuals with BD II [17] and UD [18]. The superior temporal resolution of ERP enables the tracking of neural activity associated with impaired cognitive performance, providing vital insights for developing therapeutic interventions [19]. Consequently, ERP components show promise as potential biomarkers for certain aspects of specific mental illnesses [20]. However, relatively few studies in recent decades have directly compared ERP components between patients with BD II and UD. While a systematic review confirmed significant P300 latency differences between bipolar disorder and UD, it highlighted a critical knowledge gap: the lack of subtype-specific data for BD II [21]. Other studies reported that patients with BD II exhibit reduced amplitudes in MMN and P300, when compared to patients with UD or healthy controls (HC) [22, 23].

Therefore, we hypothesize that patients with UD or BD II may exhibit distinct cognitive impairments and that ERP could serve as a significant biomarker for differentiating between UD and BD II. This study aimed to assess neurophysiological responses in patients with BD II, patients with UD, and matched HC, and to determine whether ERP components can function as potential electrophysiological biomarkers for distinguishing BD II from UD.

## Methods

### Sample size estimation

We aimed to investigate differences in ERP components across three groups: BD II, UD, and HC. An a priori power analysis using G*Power 3.1 [24] determined a minimum sample size of 144 participants (α = 0.05, power (1–β) = 0.90, effect size Cohen's f = 0.3, df = 2,λ = 12.9, *n* = λ/f^2^ = 12.9/0.09≈143.3) for detecting group differences via analysis of variance (ANOVA). Ultimately, 180 participants were recruited (60 per group).

### Participants and procedure

This retrospective case–control study enrolled 180 participants from March 2020 to February 2023, and all participants were stratified into three distinct groups: (1) UD group: 60 patients diagnosed with MDD; (2) BD II group: 60 patients diagnosed with BD II and currently experiencing a depressive episode; and (3) HC group: 60 age- and sex- matched healthy participants. Patients with BD II and UD were recruited through the Department of Psychiatry at Sichuan Provincial People's Hospital, while the HC group were recruited from community.

The inclusion criteria for this research were as follows: (1) Aged 18–60 years, female or male; (2) UD group patients met DSM-5 [25] diagnostic criteria for MDD with current depressive episode; (3) BD II group patients met DSM-5 [25] diagnostic criteria for bipolar II disorder with current depressive episode; (4) Healthy participants were required to have: a) No lifetime history of psychiatric disorder; b) Absence of chronic physical illnesses; (5) No history of use of antipsychotics, antidepressants, or other psychotropic medications within 4 weeks prior to baseline assessment; (6) Complete abstinence from alcohol, tobacco, and caffeine-containing products for at least 24 h before the examination; (7) Provision of written informed consent.

The exclusion criteria for this research were as follows: (1) Meeting DSM-5 [25] diagnostic criteria for other mental disorders, (e.g., bipolar I disorder, schizophrenia, obsessive–compulsive disorder, alcohol/substance use disorders); (2) Clinically significant mental or physical conditions impairing task performance; (3) Abnormal auditory brainstem response (ABR) tests (hearing threshold > 60 dB nHL) to confirm intact auditory pathways; (4) History of concussion or brain injury with loss of consciousness within 6 months; (5) Electroconvulsive therapy within 12 months; (6) Diagnosis of neurological disorders.

The study protocol was approved by the Ethics Committee of Sichuan Academy of Medical Sciences and Sichuan Provincial People's Hospital. All procedures were conducted in accordance with the World Medical Association Declaration of Helsinki.

### Psychological assessment tools

#### Patient Health Questionnaire-9 (PHQ-9)

The PHQ-9 is a validated and widely used instrument for depression screening and severity assessment in clinical settings [26, 27]. This self-report scale comprises 9 items evaluating symptom frequency over the last two weeks. Each item is rated on a 4-point Likert scale (0 = "not at all" to 3 = "nearly every day"), generating total scores from 0 to 27. Severity is stratified using established cutoff values, with scores ≥ 10 demonstrating optimal sensitivity and specificity for depression diagnosis in clinical populations [28].

#### Generalized Anxiety Disorder −7 GAD-7)

The GAD-7 is a widely used psychometric instrument assessing anxiety symptoms, with established criterion validity, factorial validity, and diagnostic validity [27, 29]. This instrument demonstrates clinical utility in both research and clinical practice, facilitating efficient screening, diagnosis, and severity assessment of anxiety disorders [29].

#### 32-item Hypomania Checklist (HCL-32)

The HCL-32 [30] is a validated self-report instrument with robust psychometric properties in diverse populations [31, 32]. This 32-item tool uses a dichotomous (yes/no) format to assess lifetime hypomanic manifestations, where yes responses are scored 1. A cutoff of ≥ 14 has been established as optimal for detecting historical hypomanic episodes in mood disorder populations [33, 34].

### ERP procedures and data analysis

The ABR testing and ERP recordings were performed by a certified neurophysiology technician with over 10 years of specialized experience. All participants underwent standardized electrophysiological assessments in a controlled environment. Patients in the BD II and UD groups were experiencing current depressive episodes at the time of testing.

All participants underwent testing while seated in a reclining chair within an acoustically shielded chamber, where ambient noise levels were maintained below 30 dB nHL throughout the procedure. Participants with abnormal auditory brainstem response (ABR) results (hearing threshold > 60 dB nHL) were excluded to confirm intact auditory pathways.

All participants completed four ERP paradigms in the following fixed sequence during a single 30-min session: (1) MMN (pre-attentive deviant detection in a passive oddball paradigm), (2) P300 paradigm (target stimulus detection in an auditory oddball task), (3) CNV (anticipatory response to warning tones), (4) P50 suppression (paired-click sensory gating assessment).

Electroencephalogram (EEG) data were required using silver/silver-chloride electrodes placed at Fz and Cz, referenced to the earlobes (the mastoid electrodes served as a reference for all EEG electrode channels online). The Fpz electrode served as ground. Data collection employed a Neuropack MEB-9200 evoked potential system (Nihon Kohden, Japan) coupled to an auditory stimulator.

Signal averaging was performed with 0.1–100 Hz bandpass filtering. Epochs exceeding ± 300 μV in amplitude were automatically rejected. The analysis time windows were paradigm-specific: For MMN and P300: −100 to 1000 ms relative to stimulus onset (pre-stimulus baseline correction). For CNV, −500 to 5000 ms. For P50 suppression, 0 to 600 ms. Following filtering, data were resampled to 1000 Hz. The amplifier specifications were listed as follows: input impedance: ≥ 1000 MΩ, Noise: < 0.6 μV rms (1-10 k Hz bandwidth, input short-circuited), Common mode rejection ratio: ≥ 112 dB. Low-cut filter was 0.1 Hz at 6 dB/oct for MMN and P300 paradigms, and 0.01 Hz at 6 dB/oct for CNV and P50. High-cut filter was set as follow: 50 Hz at 6 dB/oct for MMN, 100 Hz at 12 dB/oct for P300, 20 Hz at 12 dB/oct for CNV, and 1000 Hz at 12 dB/oct for P50. Alternating current interference notch filter: 50 Hz (rejection ratio < 1/20); and skin–electrode contact impedance check: < 5 kΩ. The data were processed using overlapping averaging methods.

#### MMN paradigm

The MMN paradigm comprised 200 auditory stimuli administered in a pseudorandom sequence. The stimulus set included two categories: rarely ‘deviant’ stimuli (20% probability; 100 ms ‘da’, 80 dB nHL) and frequently ‘standard’ stimuli (80% probability; 100 ms ‘da’, 60 dB nHL), with an inter-stimulus interval (ISI) of 1000 ms between consecutive stimuli. Explicit instructions emphasized passive listening without active sound discrimination. Epochs contaminated by eye blinks were excluded from ERP averaging, with additional artifact-free deviant trials added to maintain a minimum of 200 valid trials per subject. The key metrics of MMN components were MMN latency (time interval between the onset of deviant stimulus and the peak negativity of the MMN components) and MMN amplitude (the voltage difference between the MMN peak negativity and the pre-stimulus baseline).

#### P300 paradigm

The P300 paradigm employed a two-tone auditory oddball task with 30–36 deviant stimuli serving as targets interspersed among standard stimuli, delivered binaurally via headphones. All stimuli consisted of 100 ms tones presented at 1 Hz, with standard stimuli at 60 dB nHL and deviant stimuli at 80 dB nHL. EEG signals were acquired using a 0.1–100 Hz bandpass filter and epochs exceeding ± 40 μV amplitude were excluded from analysis. Participants were instructed to press a response button immediately upon detecting deviant stimuli.

The P300 encompasses four principal components: (1) N1, a negative deflection peaking at 80–120 ms post-stimulus; (2) P2, a positive peak maximal at 150–200 ms; (3) N2, a negative wave occurring at 200–250 ms; and (4) P3, a late positive component with 300–600 ms latency. Four composite amplitudes were quantified using peak-to-trough/trough-to-peak measurements:

- N1-P2 amplitude: from N1 trough to P2 peak;
- P2-N2 amplitude: from P2 peak to N2 trough;
- N2-P3 amplitude: from N2 trough to P3 peak;
- P2-P3 amplitude: from P2 peak to P3 peak.

#### CNV paradigm

The CNV paradigm consisted of trial sequences initiated by a warning tone (S1; 85 dB nHL). Following a fixed 2000 ms foreperiod, an imperative visual stimulus (S2; repetitive light flashes) appeared. Participants were required to immediately terminate the S2 flashes via dominant-hand button press upon stimulus detection. Each button press triggered a 10-s inter-trial interval (ITI) before subsequent S1 presentation. The CNV amplitude was calculated by averaging seven artifact-free trials.

The key metrics in the CNV paradigm were as follows:

- Point A: The onset latency of the early CNV component, which captures transition from sensory processing to anticipatory attention [35].
- Point B: The peak latency of the late CNV component, which marks peak motor readiness potential [36].
  - A-B Interval: Time span (ms) between Point A and Point B.
  - A-B Area: Total voltage activity (μV·ms) between Point A and Point B.
  - A-B Amplitude: Amplitude difference (μV) between Point A and Point B.

#### P50 suppression paradigm

The sensory gating paradigm employed paired auditory clicks (S1: conditioning stimulus; S2: test stimulus) at 80 dB nHL presented binaurally through headphones. The S1-S2 interval was 500 ms (inter-stimulus interval), and the ITI was 10 s. The subjects were instructed to passively listen, and maintain central fixation on a crosshair without overt behavioral response. Sixteen artifact-free trials were averaged to generate composite waveforms for analysis purposes. Grand averages for S1 and S2 were computed separately using a weighted averaging algorithm.

The key metrics in the P50 suppression paradigm were as follows.

- A1 amplitude: The peak-to-baseline voltage difference (µV) of the P50 component evoked by the S1.
- A2 amplitude: The peak-to-baseline voltage difference (µV) of the P50 component evoked by the S2.
- S1-P50 latency: The time interval (ms) from the onset of S1 to the peak of A1.
- S2-P50 latency: The time interval (ms) from the onset of S2 to the peak of A2.

### Statistical analyses

Statistical analyses were performed using SPSS 26.0 (IBM Corp) for parametric and non-parametric analyses, and GraphPad Prism 9.0 for scientific graphing. Chi-square tests were applied to categorical variables. Continuous variables were presented as mean ± standard deviations (SD), while categorical variables were reported as frequencies and percentages. Normality was assessed using the Kolmogorov–Smirnov test [37]. To establish baseline comparability, demographic and clinical characteristics across the three groups (HC, BD II, UD) were formally tested using: (1) χ^2^ tests (or Fisher's exact test) for categorical variables; (2) One-way ANOVA (normally distributed) or Kruskal–Wallis test (non-normal) for continuous variables [38]. Bonferroni post hoc correction was performed for multiple comparisons to reduce the risk of type I error [38]. ANCOVA models (SPSS GLM procedure) were constructed with diagnosis group (UD = 1, BD II = 2) as a fixed factor, and age [39], sex (dummy-coded 0/1) [40], and GAD-7 total score [41] as covariates, based on previous studies suggesting their potential impact on ERP components in depression patients. Pearson correlation (normally distributed variables) or Spearman’s rank order correlation (non-normal variables) analyses were performed to assess associations between ERP components and PHQ-9 total score within the UD and BD II groups separately. Benjamini–Hochberg false discovery rate (FDR) correction was subsequently implemented, with significant associations determined at q < 0.05. In addition to statistical significance, effect size estimates were reported alongside inferential statistics, including standardized β coefficients and odds ratios (OR) with 95% confidence intervals (95% CI) for association analyses. The diagnostic accuracy of the logistic regression model was evaluated by receiver operating characteristic (ROC) curve analysis using GraphPad Prism 9.0. An area under the curve (AUC) > 0.7 was considered clinically meaningful [42]. All analyses used two-sided tests with α = 0.05 defining statistical significance. Exact *P*-values were reported to three decimal places, with values below 0.001 denoted as *P* < 0.001. All reported *P*-values for group comparisons were adjusted for covariates.

## Results

### Sociodemographic characteristics

A total of 180 participants were enrolled into this study. All subjects were categorized into three groups: BD II (*n* = 60), UD (*n* = 60), and HC (*n* = 60). For the UD, BD II, and HC groups, the mean age was 38.0 ± 13.0, 36.1 ± 11.9, and 34.1 ± 7.6 years (F = 1.757,* P* = 0.176), and the proportion of female sex was 76.7%, 80.0%, and 70.0% (χ^2^ = 1.684, *P* = 0.431), respectively. Other demographic characteristics, including education, marital status, and socioeconomic status, showed no significant differences across groups (all* P* > 0.05). The total scores of the GAD-7 and PHQ-9 were significantly higher in the BD II and UD groups than in the HC group; the HCL-32 total score was significantly higher in the BD II group than in both the UD and HC groups (all *P* < 0.05) (Table 1).

**Table 1:** Sociodemographic data for the three groups of participants

Sociodemographic data	HC group (*n* = 60)	UD group (*n* = 60)	BD-II group (*n* = 60)	Contrasts	*P* value
Age (year) mean ± SD	38.0 ± 13.0	36.1 ± 11.9	34.1 ± 7.6	F = 1.757	0.176
Sex				χ2 = 1.684	0.431
Female	46 (76.7%)	48 (80.0%)	42 (70.0%)		
Male	14 (23.3%)	12 (20.0%)	18 (30.0%)		
Educational level				χ2 = 6.288	0.392
Illiteracy/primary school	7 (11.7%)	6 (10.0%)	4 (6.7%)		
Middle school	6 (10.0%)	14 (23.3%)	11 (18.3%)		
High school	10 (16.7%)	13 (21.7%)	14 (23.3%)		
Bachelor degree or higher	37 (61.7%)	27.0 (45.0%)	31 (51.7%)		
Marital status				χ2 = 3.616	0.460
Unmarried	25 (41.7%)	20 (33.3%)	25 (41.7%)		
Married	33 (55.0%)	33 (55.0%)	30 (50.0%)		
Divorced/widowed	2 (3.3%)	7 (11.7%)	5 (8.3%)		
Socioeconomic status				χ2 = 0.393	0.822
Employed	44 (73.3%)	45 (75%)	42 (70%)		
Unemployed	16 (26.7%)	15 (25%)	18 (30%)		
PHQ-9 total score	1.1 ± 1.2	17.6 ± 5.5	18.8 ± 4.2	F = 346.355	< 0.001*
GAD-7 total score	2.7 ± 1.1	10.6 ± 5.5	9.7 ± 4.6	F = 62.161	< 0.001*
HCL-32 total score	3.7 ± 0.9	4.1 ± 1.1	22.4 ± 2.0	F = 3238.603	< 0.001*

*GAD-7* 7 item generalized anxiety disorder, *PHQ-9* Patient Health Questionnaire-9, *HCL-32* The 32-item Hypomania Checklist, *HC* Healthy control, *UD* Unipolar depression, *BD-II* Bipolar II depression

^*^P < 0.05 was statistically significant

### Similarities and differences in ERP components among the three groups

**Fig. 1:**
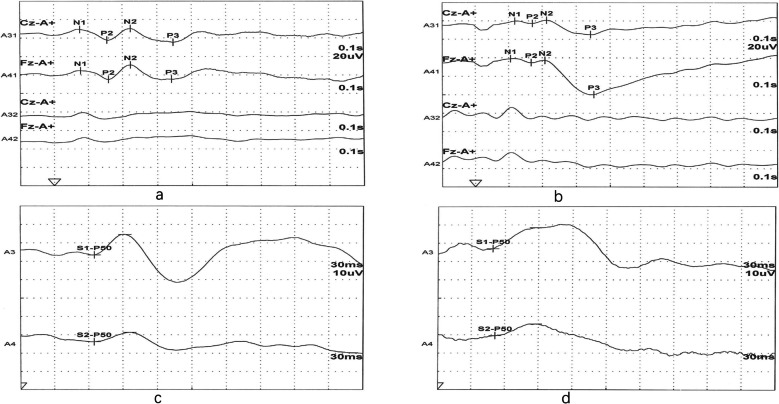
Differences in event-related potentials among patients with bipolar II depression (BD II), unipolar depression (UD), and healthy controls (HC). Both UD and BD II patients (**a**) showed larger amplitude of P2-N2 complex compared to HC (**b**). The latency of S2-P50 in BD II patients (**c**) was significantly longer than UD patients (**d**)

Table 2 and Fig. 1 showed similarities and differences in ERP components among the three groups. Compared with HC, both UD and BD II groups showed significantly longer reaction times (HC: 254.4 ± 43.8 ms; UD: 297.7 ± 72.2 ms; BD II: 300.3 ± 70.0 ms; *P* = 0.028) and larger amplitude of P2-N2 complex in the P300 paradigm (HC: 5.7 ± 4.4 μV; UD: 8.1 ± 4.8 μV; BD II: 8.6 ± 5.6 μV; *P* = 0.001) (all *P* < 0.05, Fig. 1a, b), whereas comparisons between the UD and BD II groups revealed no significant differences in these measures.

**Table 2:** Similarities and differences in ERP components among the three groups

ERP variables	HC group	UD group	BD-II group	F/H	*P* value	*P*	Post Hoc Pairwise* P*-values (*P*^a^)		
							HC vs.UD	HC vs. BD-II	UD vs. BD-II
MMN									
MMN latency (ms)	218.2 ± 22.5	216.6 ± 34.1	211.4 ± 29.0	0.904	0.407	0.731	1.000	0.599	0.987
MMN amplitude (uV)	4.2 ± 3.2	4.1 ± 3.8	4.0 ± 3.1	0.012	0.988	0.084	1.000	1.000	1.000
P300									
Button press count	31.3 ± 1.6	30.9 ± 1.9	30.9 ± 1.4	1.162	0.315	0.161	0.591	0.545	1.000
Reaction time (ms)	254.5 ± 43.8	297.7 ± 72.2	300.3 ± 70.0	9.827	< 0.001*	0.028*	0.001*	< 0.001**	1.000
N1 latency (ms)	100.1 ± 19.5	97.6 ± 19.1	95.8 ± 18.9	0.763	0.468	0.788	1.000	0.662	1.000
P2 latency (ms)	160.1 ± 23.3	162.4 ± 20.9	158.0 ± 21.9	0.610	0.545	0.893	1.000	1.000	0.813
N2 latency (ms)	205.2 ± 16.5	213.2 ± 17.3	216.2 ± 22.1	5.446	0.005*	0.044*	0.066	0.005*	1.000
P3 latency (ms)	319.6 ± 18.9	315.6 ± 29.4	310.4 ± 28.9	1.851	0.160	0.437	1.000	0.170	0.862
N1-P2 complex (uV)	8.6 ± 4.5	8.6 ± 3.9	6.7 ± 4.3	3.445	0.034*	0.116	1.000	0.067	0.070
P2-N2 complex (uV)	5.7 ± 4.4	8.1 ± 4.8	8.6 ± 5.6	5.757	0.004*	0.001*	0.035*	0.005*	1.000
P2-P3 complex (uV)	12.0 ± 7.1	8.6 ± 11.3	6.6 ± 5.1	6.117	0.003*	0.058	0.086	0.002*	0.596
N2-P3 complex (uV)	15.5 ± 6.7	12.4 ± 8.3	10.7 ± 7.0	6.345	0.002*	0.378	0.075	0.002*	0.642
CNV									
Reaction time (ms)	2178.9 ± 196.0	2262.3 ± 234.6	2212.1 ± 193.5	2.403	0.093	0.288	0.092	1.000	0.574
Point A latency (ms)	416.5 ± 207.5	350.1 ± 159.1	342.1 ± 196.7	2.793	0.064	0.143	0.172	0.097	1.000
Point B latency (ms)	1430.6 ± 380.3	1267.9 ± 397.7	1330.9 ± 447.6	2.409	0.093	0.331	0.093	0.552	1.000
A-B interval (ms)	1014.0 ± 383.8	974.2 ± 433.1	1085.1 ± 580.4	0.833	0.436	0.888	1.000	1.000	0.611
A-B amplitude(uV)	23.3 ± 8.2	18.7 ± 10.4	21.5 ± 8.4	3.868	0.023*	0.126	0.119	0.869	0.306
A-B area (uVs)	13.1 ± 8.6	9.3 ± 7.3	12.1 ± 9.8	3.001	0.052	0.227	0.058	1.000	0.257
P50									
S1-P50 latency (ms)	55.8 ± 12.2	55.3 ± 13.2	58.8 ± 11.8	1.285	0.279	0.105	1.000	0.597	0.424
S2-P50 latency (ms)	57.1 ± 13.4	50.4 ± 11.1	63.2 ± 11.5	6.643	0.002*	0.025*	0.153	0.287	< 0.001**
Amplitude A1 (uV)	13.4 ± 8.1	11.8 ± 7.3	11.2 ± 5.9	1.488^a^	0.475	0.884	0.329	0.266	0.855
Amplitude A2 (uV)	6.6 ± 3.2	7.6 ± 3.8	8.3 ± 4.8	3.009^a^	0.222	0.094	0.161	0.120	0.726

*MMN* Mismatch negativity, *CNV* Contingent negative variation, *ERP* Event-related potential, *HC* Healthy control, *UD* Unipolar depression, *BD-II*: Bipolar II depression

^*^P < 0.05 was statistically significant

^**^P < 0.001 was statistically significant

*P*: *P*-values derived from analysis of covariance (ANCOVA) adjusting for age, sex, and GAD-7 total score

*P*^a^: *P*-values derived from Bonferroni multiple comparison correction

F value derived from ANOVA test

^a^H value derived from Kruskal–Wallis H test

In addition, the S2-P50 latency in the BD II group was significantly longer than the UD group (UD: 50.4 ± 11.1 ms vs. BD II: 63.2 ± 11.5 ms; *P* = 0.025), and the N2 latency in the BD II group was longer than the HC group (BD II: 216.2 ± 22.1 ms vs. HC: 205.2 ± 16.5 ms; *P* = 0.044) (both *P* < 0.05, Fig. 1c, d).

Due to non-normal distribution, amplitudes A1 and A2 were compared among the three groups using the Kruskal–Wallis H test. No significant differences were observed in either amplitude (all *P* > 0.05).

### Analysis of ERP indices for discriminating UD and BD II.

**Fig. 2:**
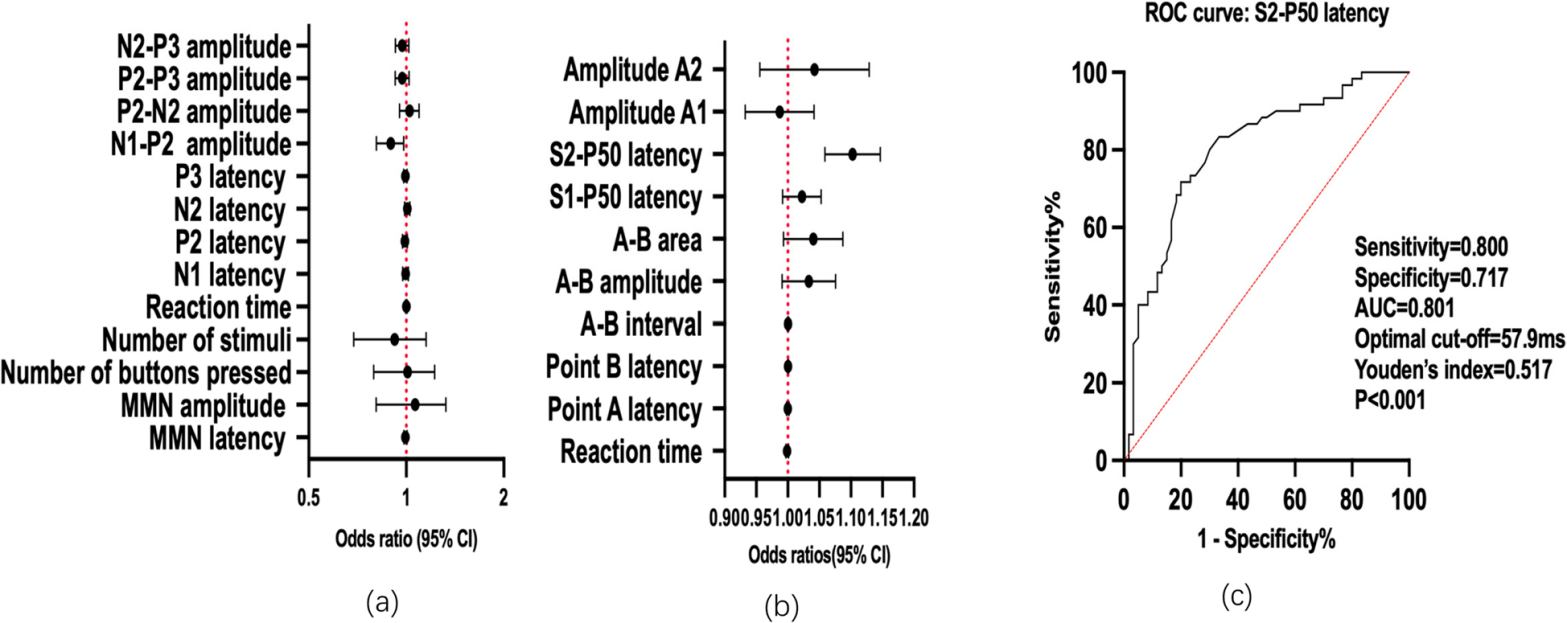
Univariate and ROC analyses for differentiating bipolar II depression (BD II) from unipolar depression (UD). **a** MMN and P300 components for differentiating BD II from UD: Univariate odds ratios. **b** CNV and P50 components for differentiating BD II from UD: Univariate odds ratios. **c** ROC curve analysis of S2-P50 latency for differentiating BD II from UD (AUC = 0.801)

As shown in Table 3 and Fig. 2, logistic regression identified S2-P50 latency as a significant discriminator of BD II from UD (β = 0.097, *P* = 0.020; OR = 1.102, 95% CI:1.059–1.147). ROC analysis demonstrated significant diagnostic utility for differentiating BD II from UD, with an AUC of 0.800 (95% CI: 0.720–0.880; *P* < 0.001). At the optimal cut-off of 57.9 ms of S2-P50 latency, sensitivity reached 0.800 and specificity 0.717.

**Table 3:** Logistic regression analysis of ERP indices for discriminating UD and BD-II

Variables	β	Standard error	Wards	*P*	OR	95% CI (lower limit, upper limit)		*P*	AUC
MMN									
MMN latency (ms)	−0.005	0.006	0.796	0.372	0.995	0.983	1.006	0.571	
MMN amplitude (uV)	−0.002	0.099	0.000	0.987	0.998	0.823	1.212	0.971	
P300									
Button press count	−0.006	0.110	0.003	0.954	0.994	0.802	1.232	0.939	
Reaction time (ms)	0.001	0.003	0.040	0.842	1.001	0.995	1.006	0.502	
N1 latency (ms)	−0.005	0.010	0.266	0.606	0.995	0.976	1.014	0.448	
P2 latency (ms)	−0.010	0.009	1.285	0.257	0.990	0.973	1.007	0.319	
N2 latency (ms)	0.008	0.009	0.674	0.412	1.008	0.989	1.026	0.402	
P3 latency (ms)	−0.006	0.006	0.917	0.338	0.994	0.981	1.006	0.427	
N1-P2 complex (uV)	−0.113	0.050	5.178	0.023*	0.893	0.810	0.984	0.033*	ROC = 0.362, *P* = 0.013
P2-N2 complex (uV)	0.022	0.035	0.383	0.536	1.022	0.954	1.096	0.969	
P2-P3 complex (uV)	−0.029	0.025	1.346	0.246	0.972	0.925	1.020	0.247	
N2-P3 complex (uV)	−0.029	0.025	1.421	0.233	0.971	0.926	1.019	0.119	
CNV									
Reaction time (ms)	−0.001	0.001	1.591	0.207	0.999	0.997	1.001	0.168	
Point A latency (ms)	0.000	0.001	0.062	0.804	1.000	0.998	1.002	0.978	
Point B latency (ms)	0.000	0.000	0.668	0.414	1.000	1.000	1.001	0.283	
A-B interval (ms)	0.000	0.000	1.375	0.241	1.000	1.000	1.001	0.183	
A-B amplitude (uV)	0.032	0.021	2.354	0.125	1.033	0.991	1.076	0.133	
A-B area (uVs)	0.039	0.023	2.816	0.093	1.039	0.994	1.088	0.090	
P50									
S1-P50 latency (ms)	0.022	0.015	2.102	0.147	1.022	0.992	1.053	0.212	
S2-P50 latency (ms)	0.097	0.020	23.071	< 0.001**	1.102	1.059	1.147	< 0.001**	ROC = 0.800, *P* < 0.001**
Amplitude A1 (uV)	−0.014	0.028	0.258	0.612	0.986	0.933	1.042	0.389	
Amplitude A2 (uV)	0.039	0.042	0.847	0.358	1.040	0.957	1.130	0.563	

*CI* Confidential interval, *MMN* Mismatch negativity, *CNV* Contingent negative variation, *ERP* Event related potential, *UD* Unipolar depression, *HC* Healthy controls, BD-II: Bipolar II depression, *AUC* Area under the receiver operating characteristic (ROC) curve

^*^P < 0.05 was statistically significant

^**^P < 0.001 was statistically significant

*P*: *P*-values derived from Logistic regression analysis adjusting for age, sex, and GAD-7 total score

Bivariate logistic regression analysis revealed that reduced amplitude of the N1-P2 complex was associated with BD II diagnosis (β = − 0.113, *P* = 0.023; OR = 0.893, 95% CI: 0.810–0.984). In contrast, ROC curve analysis demonstrated no diagnostic capacity for this measure, yielding an AUC of 0.362 (95% CI: 0.257–0.468).

No other ERP parameters showed statistically significant differences between BD II and UD in adjusted logistic regression models (all *P* > 0.05).

### Correlation between ERP components and PHQ-9 total score

Pearson correlation analyses revealed no significant associations between any ERP component and PHQ-9 total score in either the UD group (*q* = 0.44–0.98, |r|= 0.01–0.25) or BD II group (*q* = 0.28–0.98, |r|= 0.01–0.39) following FDR correction.

## Discussion

Our study found that BD II patients exhibited prolonged S2-P50 latency compared to UD patients. To our knowledge, this study provides novel evidence that S2-P50 latency may assist in differentiating BD II from UD in clinical practice. P50 is an effective tool to measure sensory gating function [43], which reflects a neural filtering mechanism that suppresses redundant or non-salient sensory inputs without conscious effort [44]. Sensory gating deficits manifest as excessive influx of irrelevant sensory information, impaired selective attention and information processing, alongside cognitive decline [45]. One study reported prolonged S2-P50 latency in schizophrenia patients, which correlated positively with cognitive impairment [46]. Another study proposed that P50 latency could serve as a clinical biomarker for identifying individuals at elevated risk of measurable cognitive decline [47]. Our study indicates that BD II patients exhibit more severe sensory gating deficits than UD patients, as evidenced by prolonged S2-P50 latency. Given the scarcity of historical research on P50 latency in BD II and UD, future studies are needed to validate the utility of S2-P50 latency in distinguishing between BD II and UD.

Our analysis confirmed that S2-P50 latency demonstrated significant diagnostic value for differentiating BD II from UD. The ROC-derived AUC of 0.800 (95% CI: 0.720–0.880) exceeded the threshold for clinically useful discrimination (AUC > 0.70) [42]. At the optimal cut-off of 57.9 ms — determined by optimizing sensitivity and specificity trade-offs — this measure achieved a sensitivity of 0.800 (correctly identifying 80% of BD II cases) and a specificity of 0.717 (correctly excluding 71.7% of UD cases). These findings suggested that prolonged S2-P50 latency may reflect underlying neurophysiological distinctions between BD II and UD.

In the P300 paradigm, both BD II and UD showed significantly prolonged reaction time compared to healthy controls; although no significant difference was observed between the BD II and UD groups. These findings suggest that impairments in two stages of information processing — stimulus assessment and motor reaction initiation — might represent a core cognitive deficit during the depressive phase across the mood disorder spectrum. This slowed information processing might be related to altered global connectivity within the frontal-parietal cognitive control network, which has been linked to depressive symptoms [48]. Consequently, these impairments might partly account for the psychomotor retardation characteristic of depressive states, affecting both BD II and UD. Moreover, the lack of significant differences between BD II and UD in this paradigm supported the notion that impairment in basic cognitive processing (indexed by P300 reaction time) was predominantly mediated by the depressive state, rather than diagnostic categories (BD II vs. UD).

Our study found that larger P2-N2 amplitudes in both the UD and BD II groups compared to healthy participants. P2 indexes higher-order cognitive mechanisms extending beyond sensory encoding, whereas N2 corresponds to attentional allocation and conflict monitoring processes [49] and the amplitude of P2-N2 complex may reflect a continuum from sensory integration to cognitive control, and abnormalities may indicate broader cognitive dysfunction. These findings suggest that both UD and BD II patients have severe cognitive impairments. However, the lack of significant difference between BD II and UD was inconsistent with previous studies [50], which may suggest similar cognitive impairment between BD II and UD patients. Heterogeneity in study populations across different studies may partly account for this discrepancy. Most studies fail to rigorously distinguish between depressive episodes and other illness phases (e.g., manic or mixed states) in bipolar II disorder, potentially overestimating the specificity of impairments during acute depressive episodes [49]. More studies are necessary to expand the knowledge about the clinical and neuropathological significance of abnormalities in the amplitude of P2-N2 complex in UD and BD II patients.

In addition, significantly prolonged N2 latency was observed in BD II patients compared with healthy controls. This prolonged N2 latency was correlated with impairments of conflict inhibition [51], indicating potential dysfunction in executive control mechanisms in BD II patients. Although a prior study observed prolonged N2 latency in bipolar disorder, critical details – such as bipolar subtype specification – were not reported, thereby limiting direct comparability with our findings [49]. Therefore, the generalizability of our findings requires further validation due to heterogeneity across existing research in study populations and task paradigms.

### Limitations

It is necessary to consider the following limitations when interpreting the findings. First, recruiting participants exclusively from Sichuan Province, may limit the generalizability of the findings to populations with diverse ethnic and geographical backgrounds across China. Therefore, future larger-scale, multi-center studies with ethnically diverse samples are warranted to enhance the reliability and validity of the findings. In addition, the ERP paradigms used in the study may not fully reflect the complex cognitive functions observed in real-world scenarios. Therefore, future research should aim to develop and utilize ERP paradigms with higher ecological validity to better capture cognitive processes in naturalistic settings. Furthermore, we utilized self-report scales as the primary instrument to assess symptom severity. In future investigations, clinician-rated scales, such as the Hamilton Anxiety Rating Scale (HAMA) and Hamilton Depression Rating Scale (HAMD), should be used to enhance the reliability and validity of the findings.

## Conclusions

This study may identify neurophysiological distinctions between BD II and UD depression, notably a prolonged S2-P50 latency in BD II.

## Data Availability

The data that support the findings of this study are not openly available due to reasons of sensitivity and are available from the corresponding author upon reasonable request. Data are located in controlled access data storage at Mental Health Center of Sichuan Provincial People's Hospital.

## Abbreviations

ABR: Auditory brainstem response
AUC: Area under the curve
BD II: Bipolar II disorder
CNV: Contingent negative variation
DSM-V: Diagnostic and Statistical Manual of Mental Disorders
ERP: Event-related potentials
FDR: False discovery rate
GAD-7: Generalized Anxiety Disorder -7
HC: Healthy controls
HCL-32: 32-Item Hypomania Checklist
ICD-11: International Classification of Diseases
ITI: Inter-trial interval
HAMA: Hamilton Anxiety Rating Scale
HAMD: Hamilton Depression Rating Scale
MDD: Major depressive disorder
MMN: Mismatch negativity
OR: Odds ratios
PHQ-9: Patient Health Questionnaire-9
SD: Standard deviations
UD: Unipolar depression
